# Supplementary material for: Favorable Vasomotor Function after Drug-Coated Balloon-Only Angioplasty of De Novo Native Coronary Artery Lesions
Source: J Clin Med. 2022 Jan 7;11(2):299. doi: 10.3390/jcm11020299 (PMC8779419; doi:10.3390/jcm11020299)

SUPPLEMENATARY MATERIAL

Favorable vasomotor function after drug-coated balloon angioplasty of *de novo* native coronary arteries

Sunwon Kim, MD, PhD; Jong-Seok Lee, MD; Yong-Hyun Kim, MD, PhD; Jin-Seok Kim, MD, PhD; Sang-Yup Lim, MD, PhD; Seong Hwan Kim, MD, PhD; Minjung Kim; Jeong-Cheon Ahn, MD, PhD; and Woo-Hyuk Song, MD, PhD

Cardiovascular Center, Korea University Ansan Hospital, Ansan-si, South Korea

Supplementary Table S1. Clinical characteristics of the study population

	Patients (n = 132)
Age (years)	62.1 ± 10.1
Male	100 (75.8 %)
Height (cm)	163.9 ± 8.9
Weight (kg)	67.2 ± 11.0
Body mass index	24.9 ± 2.8
Risk factors	
Smoking	38 (28.8 %)
Hypertension	86 (65.2 %)
Diabetes	50 (37.9 %)
Dyslipidemia	87 (65.9 %)
Previous stroke	4 (3.0 %)
Previous myocardial infarction	19 (14.4 %)
Previous percutaneous coronary intervention	32 (24.2 %)
Familial history of early cardiovascular disease	23 (17.4 %)
Left ventricular ejection fraction	56.8 ± 8.8
Clinical presentation	
STEMI	17 (12.9 %)
NSTEMI	22 (16.7 %)
Unstable angina	41 (31.1 %)
Stable angina	52 (39.4 %)
Laboratory findings	
Total cholesterol	154.8 ± 44.4
Triglyceride	142.9 ± 88.3
High-density lipoprotein	43.8 ± 10.7
Low-density lipoprotein	94.8 ± 43.3
Follow-up low-density lipoprotein	55.5 ± 18.9
HbA1c	6.48 ± 1.26
High sensitivity C-reactive protein	0.44 ± 1.07
Post-angioplasty medication	
Aspirin+ Clopidogrel or Ticagrelor	116 (87.9 %) or 16 (12.1 %)
Statin or Statin/Ezetimibe	20 (15.2 %) or 111 (84.8 %)
Calcium channel blocker	65 (49.2 %)
ACEi or ARB	33 (25.0 %) or 55 (41.7 %)
Beta-blocker	89 (67.4 %)
Nitrates	37 (28.0 %)

Values are expressed as means ± standard deviations or counts and percentages. ACEi = angiotensin converting enzyme inhibitor; ARB = angiotensin receptor blocker

Supplementary Figure S1. Representative case showing the three comparative arterial segments. In this patient who underwent DCB angioplasty for mid left circumflex artery lesion, proximal left anterior descending artery and big diagonal branch served as angiographically normal and spastic segments, respectively. DCB, drug-coated balloon

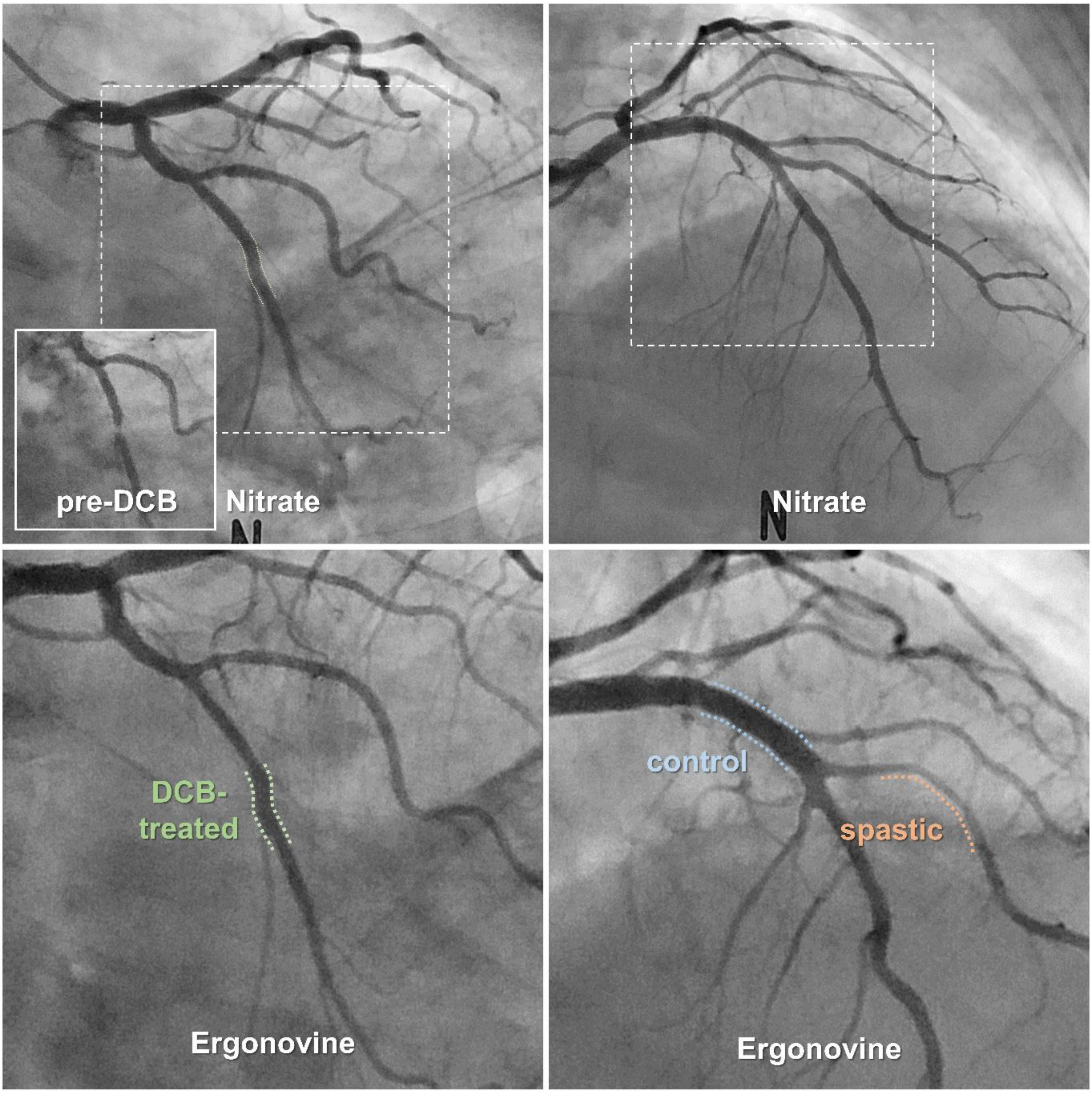

Supplementary Figure S2. A case showing a significant ergonovine-induced multifocal vasospasm. This patient experienced several episodes of alcohol-related nocturnal chest pain, which was relieved by taking calcium-channel blocker and nitrates as well as discontinuing alcohol consumption. Note that the diffuse multifocal vasospasm (red arrowheads) did not involve the DCB-treated culprit site (yellow arrowheads).

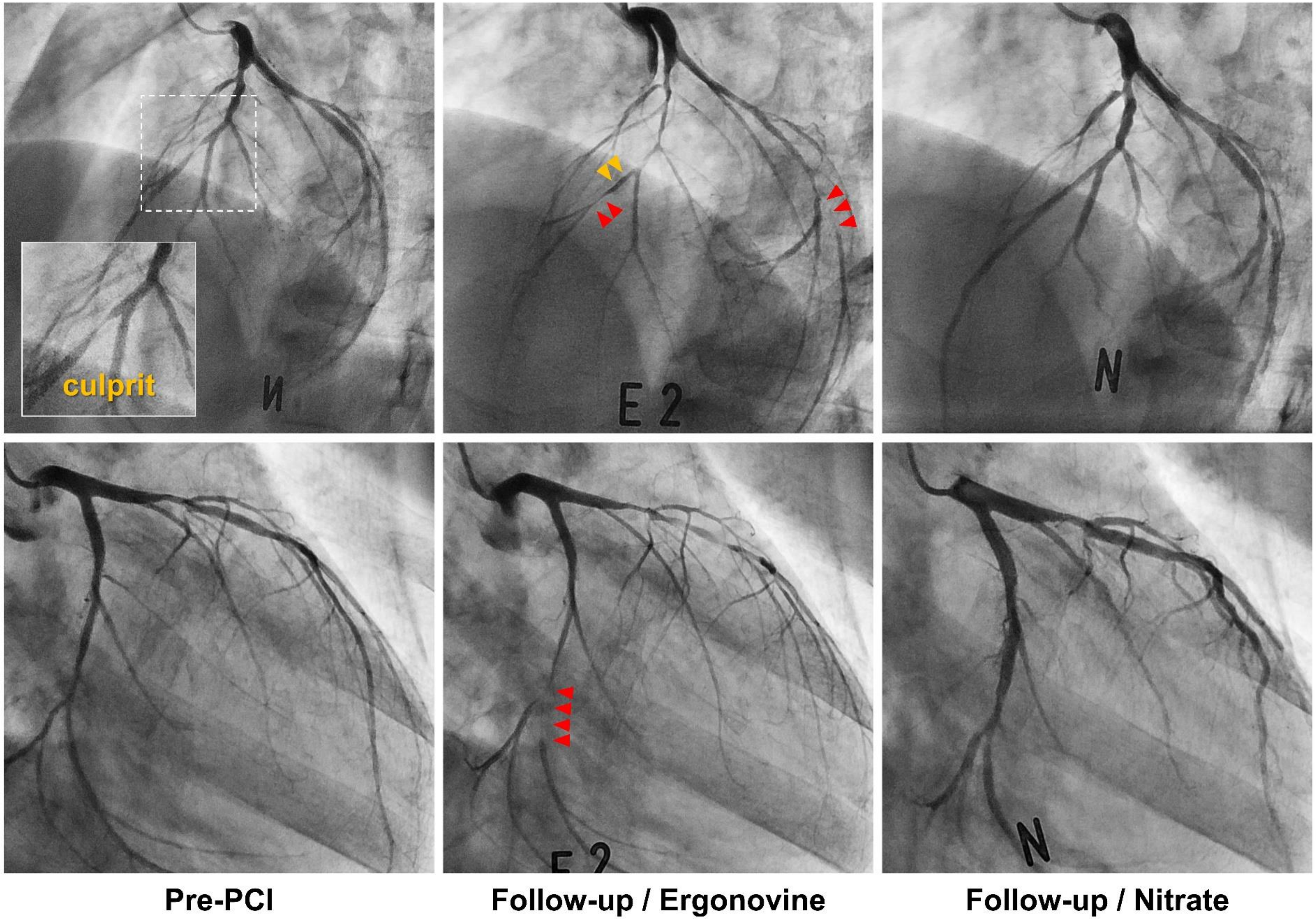

Supplementary Figure S3. Changes in the minimal lumen diameter before and after administration of ergonovine and isosorbide dinitrate.

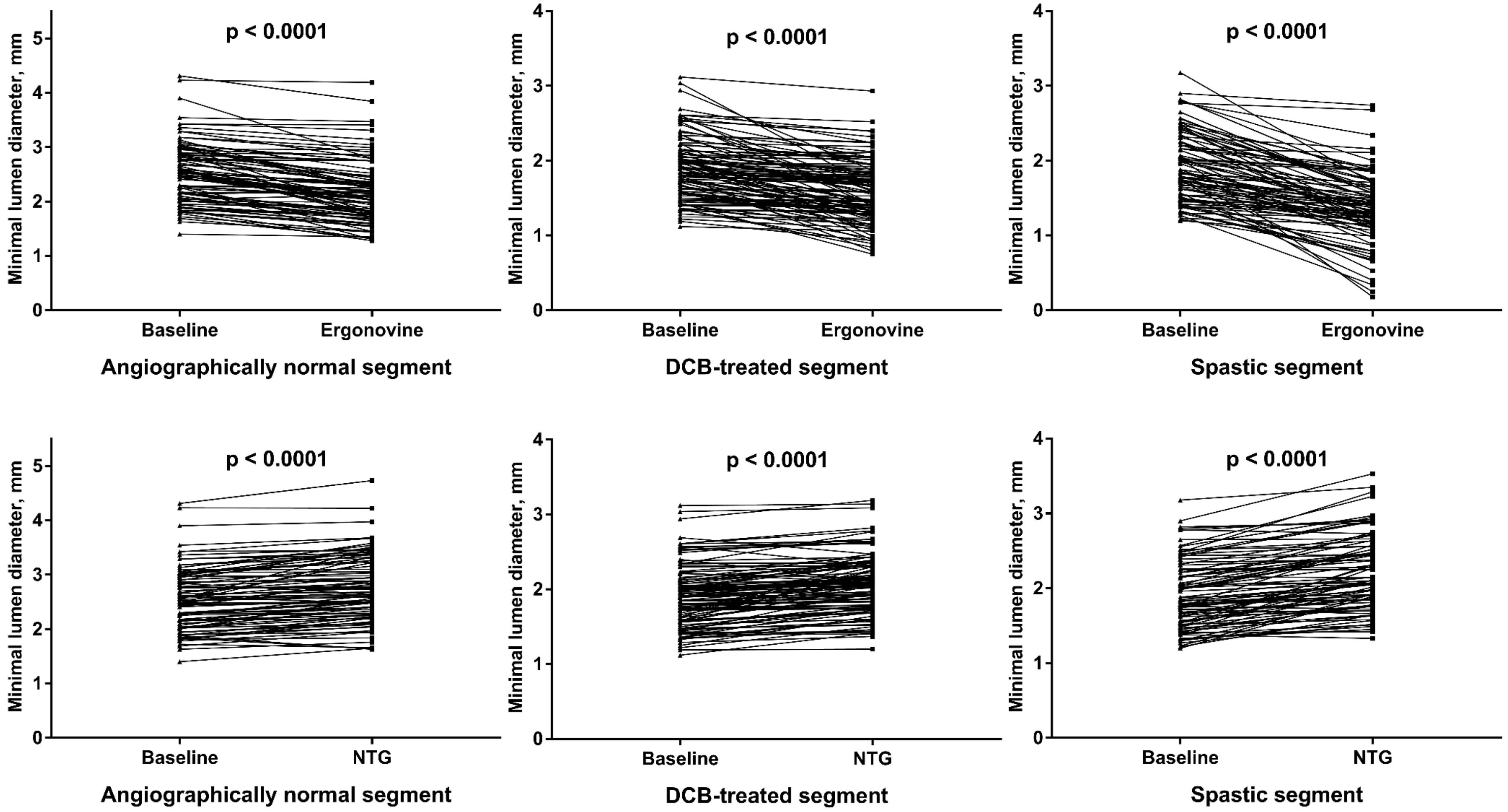

Supplement: Supplementary file 1 [file jcm-11-00299-s001.zip › jcm-1523299-supplementary.pdf]
